# Effectiveness of the 5A Counseling Model-Based Interventions on Physical Activity Indicators in Adults: A Systematic Review

**DOI:** 10.3390/bs13060476

**Published:** 2023-06-06

**Authors:** Paulo Henrique Guerra, Letícia Aparecida Calderão Sposito, Filipe Ferreira da Costa, Rogério César Fermino, Camila Bosquiero Papini, Cassiano Ricardo Rech

**Affiliations:** 1 School of Medicine, Federal University of Fronteira Sul, Chapecó 89815-899, Brazil; 2 Post-Graduation Program in Sciences of Motricity, São Paulo State University, Rio Claro 13506-900, Brazil; sposito.ef@gmail.com; 3 Associated Post-Graduation Program in Physical Education, University of Pernambuco/Federal University of Paraíba, João Pessoa 58051-900, Brazil; filipefcosta@outlook.com; 4 Postgraduate Program in Physical Education, Federal University of Technology—Paraná, Curitiba 81310-900, Brazil; rogeriofermino@hotmail.com; 5 Department of Sports Sciences, Federal University of Triângulo Mineiro, Uberaba 38025-180, Brazil; camila.papini@uftm.edu.br; 6 Post-Graduation Program in Physical Education, Federal University of Santa Catarina, Florianopolis 88040-900, Brazil; crrech77@gmail.com

**Keywords:** physical activity, counseling, primary health care, 5A

## Abstract

Background: To identify and appraise the effectiveness of the 5A-counseling-model-based interventions on indicators of physical activity in adults. Methods: A systematic review was conducted from systematic searches in Embase, Lilacs, Pubmed, Scielo, Scopus, Sportdiscus and Web of Science, involving studies published from its inception until May 2022. To avoid potential losses, searches also were made in Google Scholar and in reference lists. The assessment of studies, data extraction, and synthesis were carried out independently by two researchers. Results: Four studies composed the synthesis, which involved people with an average age between 40 and 55 years, most of the samples being women. It was observed that counseling was carried out in conjunction with other strategies, such as drawing up an action plan, sending text messages, and offering educational material. Only one study showed a statistically significant difference between the intervention and control groups in the “daily number of steps” indicator. Conclusions: Based on available studies, 5A-counseling-model-based interventions did not reflect significant findings in relation to physical activity. However, given the potential of the model, future studies are recommended with a better description of the strategies, as well as a more robust methodology, to strengthen the evidence.

## 1. Introduction

Due to its direct relationship with the health–disease process, throughout the life cycle, physical activity is one of the priority themes of the global health agenda [1]. The benefits of physical activity are widely recognized in relation to the quality of life [2] and well-being [3], as well as the prevention of different diseases [1] and early mortality [4].

Apart from these benefits, promoting changes in physical activity is not a quick or simple task, due to its multifaceted nature [1,5]. Given the need to recognize the main strategies for increasing physical activity at a community level, Heath et al. [6] indicate the potential of behavioral and social interventions.

Other studies complement this evidence, indicating, for example, the potential of counseling for physical activity in a primary health care setting [7], given the possibility of a wide reach, as well as the fact that counseling constitutes a good cost-effective educational strategy [8,9], based on dialogue and reflection on how physical activity can be inserted into people’s lives, considering their life contexts [10,11].

However, physical activity counseling can be carried out in many ways, regarding to aspects of the theoretical basis, design, duration, implementation, and assessment [10,11]. Among these, the 5A model can be highlighted, which is based on the transtheoretical model of behavior change [12,13]. Its implementation starts from the recognition of the contexts and needs of the individuals, to guide the health care process, being structured from the questioning of the behavior (assessing), the indication of doses and benefits and/or risks (advising), shared action planning (agreeing), identification of barriers and types of support (assisting) and the follow-up and assessment (arranging) [12,13].

Previous studies suggest the potential of the 5A model in addressing other behaviors and improvements in health indicators [14,15,16]. Given its potential, the present study was carried out with the aim of identifying and summarizing the impacts and effectiveness of interventions based on the 5A counseling model on physical activity indicators in adults.

## 2. Materials and Methods

A systematic review was carried out, designed, registered on PROSPERO (CRD42022333797), developed, and reported based on previous references [17,18]. Its inclusion criteria were based on the “PICOS” structure, established as follows:

- “Population”: adults, between 18 and 59 years of age, without impeding conditions for the practice of physical activity;
- “Interventions”: the provision of 5A model counselling-based interventions about physical activity, regardless of the duration, the professional nucleus that implemented it and the support of other strategies (e.g., preparation of materials, offer of practical activities);
- “Comparators”: who preferably did not receive care, or who received standard care, without offering physical activity counseling based on the 5A model;
- “Outcome”: physical activity indicators (e.g., increase in the amount of physical activity, number of steps per day/week, proportion of people meeting the recommendation, self-efficacy for physical activity, etc.), preferably the total, or in leisure time or as a form of transport;
- “Study design”: intervention studies without restrictions regarding the presence of a control group and randomized allocation between groups.

On the other hand, the exclusion criteria were literature reviews (e.g., narrative, scoping or systematic), and other forms of publication, such as dissertations, theses, abstracts and articles published in languages other than English, Portuguese or Spanish.

To identify the studies, systematic searches were conducted in seven electronic databases (Embase, Lilacs, Pubmed, Scielo, Scopus, Sportdiscus and Web of Science), covering available studies from its inception until 16 May 2022. Initially, the search strategy was developed using the Pubmed database: (((physical activity [Text Word]) OR (exercise [Text Word])) OR (walk* [Text Word])) AND (((5A[Text Word]) OR (5As [Text Word])) OR (5A’s [Text Word])) and then adapted to the other databases. The full presentation of the systematic searches is available in Supplementary Materials File S1. In addition, to avoid the loss of relevant studies, manual searches were conducted in Google Scholar, based on the terms “physical activity”, “counseling”, and “5A”, and in the reference lists of the studies assessed using their full texts.

Studies identified by systematic searches were entered into Rayyan [19]. On the platform, duplicates were identified and removed, and titles and abstracts were also screened. Subsequently, the full texts of potential articles were downloaded and assessed, as well as having their data extracted. Assessments and data extraction processes were conducted by two researchers (LS and PG), who worked independently, with the support of a third researcher to resolve doubts and establish consensus (RF).

In the title and abstract screening, exclusions were made as soon as the first divergence was identified, according to the inclusion criteria. At this stage, we did not organize the exclusions by their reasons. On the other hand, the reasons for full-text exclusions were registered and reported in a flowchart.

Data extraction was performed in an electronic spreadsheet, divided into three tabs: (I) descriptive data of the studies (e.g., study location, mean age, sample size, percentage of women, study objective, sample characteristics), (II) methodological aspects of the studies (e.g., research design, intervention implementers, research protocol, instruments used in the assessment of physical activity), and (III) results, organized by the physical activity indicators evaluated.

The descriptive synthesis was prepared based on the logic of the extraction spreadsheet (e.g., descriptive, methodological data and results). Through meetings, the researchers evaluated—from the objectives of the present review and previous experiences with other reviews—the main information to be included in the descriptive synthesis, as well as the form of its presentation.

In regard to the effect measures, we opted for the differences in means (e.g., means and variability of pre- and post-intervention data of the intervention and control groups), to provide better comparability among the findings. In studies that did not show differences in means, data were identified, and the calculations were performed using Review Manager 5.4.1 [20]. In view of the high heterogeneity between studies, metanalysis was not planned.

The Risk of bias of included studies was assessed in an adapted version of the Effective Public Health Practice Project (EPHPP) tool [21], which assesses seven methodological domains of an intervention study: “selection bias”, “adjustment of confounding variables”, “methods used in data collection”, “withdrawals and dropouts”, “protocol used in the analysis” and “use of data imputation in the analysis”.

## 3. Results

**Figure 1:**
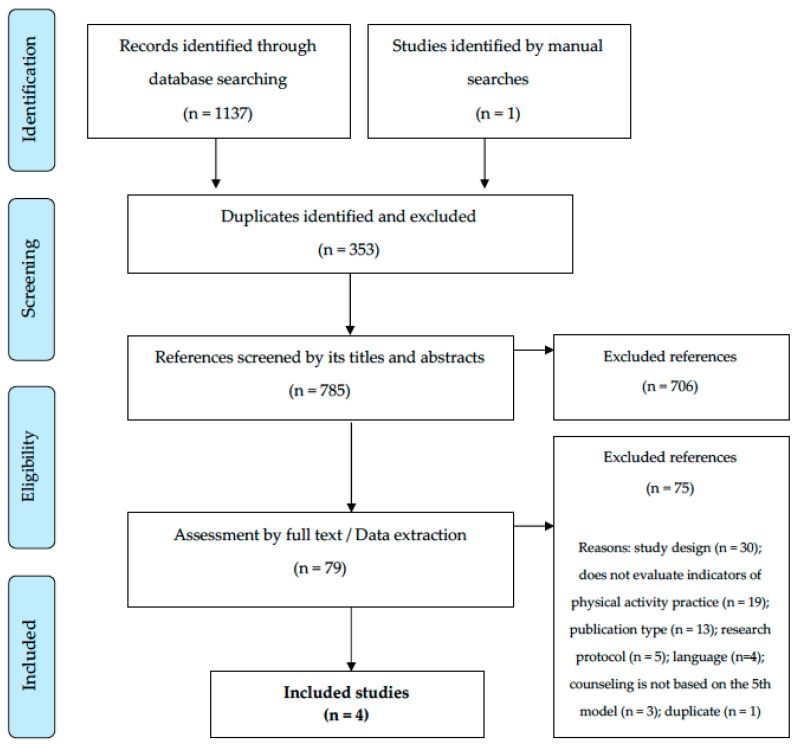
Flowchart of systematic review.

The numbers related to the review process are presented in Figure 1. Briefly, the systematic searches in the databases and manuals resulted in the identification of 1138 potential references. After removing duplicates (*n* = 353), 785 titles and abstracts were screened. From this process, 79 references remained, later assessed using their full texts. At the end of this phase, 75 references were excluded, the main reasons being the design (*n* = 30) and the “non-assessment of indicators of physical activity practice” (*n* = 19). In the end, four intervention studies adequately responded to all eligibility criteria and comprise the descriptive synthesis of the present review [22,23,24,25].

By country, two studies were carried out in the United States [23,24], one in China [25], and one in Mexico [22], involving adult populations, with a mean age between 40 [25], and 55 [24] years and higher percentage of women in three studies [22,23,25] (Table 1). Heterogeneity was observed in the profile of the participants, highlighting the involvement of inactive people/people who did not meet the recommendation [22,23], populations living in rural settings [23], overweight or obese veterans [24], and people with insomnia [25]. The objectives of the studies converged to assess the effects of interventions based on counseling on physical activity, based on the 5A model.

Except for Galaviz et al. [22], all studies presented randomization among the participants of the intervention and control groups, with heterogeneity among the implementers of the counseling, highlighting the participation of general practitioners [22], nurses [23], health students [24] and psychologists [25] (Table 2). Among the research protocols, it was observed that counseling on physical activity was offered in different settings, such as in the at routine medical consultations [22], remote/online [23,24], and highlighting other support strategies, such as the elaboration of a health action plan [23], sending messages [23] and offering educational material [24] (Table 2).

**Figure 2:**
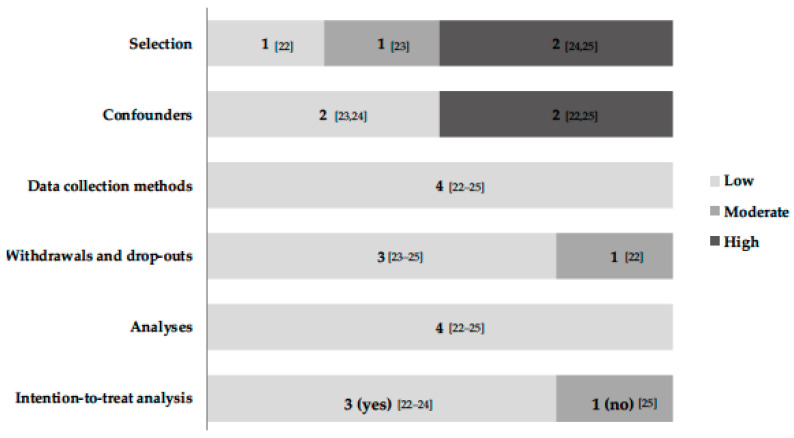
Risk of bias assessment of included studies (*n* = 4).

All studies had a low risk of bias in the domains “methods used in data collection” and “analysis protocol” (Figure 2). However, some points of high risk of bias were observed in the adjustment of confounding variables and selection of participant domains, such as (i) the lack of adjustment for the variables that showed differences between groups at baseline [22], (ii) the lack of reporting of the information evaluated [24], and (iii) populations with specific clinical conditions [24,25]. Three studies conducted the analysis with an imputation of missing data, using the “intention-to-treat” method [22,23,24].

The included studies evaluated physical activity using different instruments, such as questionnaires [22,23,24,25] and monitoring [23] (Table 3). The sample sizes analyzed varied between 43 [24] and 459 [22] participants. Heterogeneity was observed between the evaluation times of post-intervention behavior and the physical activity indicators evaluated, highlighting “number of people classified as physically active” [22], “weekly number of steps” [23], “weekly minutes” [23], and “self-efficacy for physical activity” [24]. Overall, only the study by Wang et al. [25] showed a statistically significant result between the intervention and control groups, in the indicator “daily number of steps” (2231; 95%CI = 474; 3987; *p* < 0.05).

## 4. Discussion

With the objective of identifying the effectiveness of 5A counseling model-based interventions on physical activity in adults, the present review was elaborated from four original studies, developed in three countries. In only one study was a statistically significant result observed, related to the increase in the daily number of steps [25]. In addition, heterogeneity was observed among the profile of the populations studied, research protocols, implementation processes, and physical activity indicators evaluated.

The World Health Organization suggests the promotion of physical activity in primary health care, and counseling provided by professionals has shown promising results in behavior change. For this reason, counseling is recommended as part of integrated community interventions [26]. However, the findings of the present review suggest that there is no consistent evidence supporting the positive effectiveness to recommend the implementation of model 5A in intervention studies to increase the level of physical activity. Thus, it is worth mentioning that none of the studies included involved interpersonal and environmental approaches, in parallel with the provision of counseling.

Another consideration of the results is the time for behavior consolidation. Except in Reed et al. [23], who evaluated self-efficacy for physical activity after twelve months, the other indicators of physical activity present in the synthesis were evaluated between three and six months after the start of the intervention. For example, according to the transtheoretical model of Prochaska and DiClemente [27], it is suggested that six months is the minimum time for the stability of the behavior change that involves the practice of physical activity.

In addition to what is suggested about networks and the environment, it is also worth mentioning the need for longitudinal efforts in the practice of counseling, involving different health professionals and their offer in different settings (e.g., health unit, community spaces, and home visits). Future studies can predict the insertion of 5A into a multidisciplinary work logic, considering the potential of action of different health professionals in increasing the physical activity levels [28].

It is also worth noting the reduced samples, as well as heterogeneity between the samples that participated in the studies. Two interventions were implemented in physically inactive populations, one in an urban setting and another in a rural setting and two other interventions aimed at populations with specific clinical conditions, such as obesity [24] and insomnia [25]. A previous study suggests differences in the determinants of physical activity in free time between women living in urban and rural settings [29], which reduces the comparability between studies. On the other hand, it is necessary to take a more careful look at the practice of counseling on physical activity in populations with more specific clinical conditions, based primarily on their health situation. Thus, we suggest that future research may involve larger and more heterogeneous samples, both in terms of physical activity levels and their clinical condition.

Even though it was not the objective of the present review, we consider it important to mention the lack of a more in-depth report on the application of the 5A model in the practice of counseling on physical activity. Apart from the conceptual similarity between the models used in the studies, we believe that this information is important and, in some way, can guide the practice of counseling on physical activity, given its high frequency, particularly in primary health care settings [30,31]. We also consider it important to indicate the main demands and challenges for the application of the model, taking as a suggestion, for example, the structure suggested by Alahmed and Lobelo [13].

This review has some limitations, and perhaps the main one is the reduced number of studies in the synthesis. However, we justify it in some ways, because it seems that dealing with behavioral research has this difficulty in recruiting and following people for a longer period—and this may also be one of the main challenges for evaluating the 5A in future studies. Additionally, we suggest caution in extrapolating the results, considering (i) some methodological biases found, such as the lack of adjustment for confounding factors; (ii) the presence of samples with clinical conditions (obese people with comorbidities and people with insomnia); and (iii) the greater representation of women in the samples. Recognizing that different groups have different determinants for physical activity [32,33,34], it is important that future studies adapt the design and implementation of the 5A model to contemplate such specificities.

On the other hand, we believe that the present review stands out for presenting a more specific synthesis of studies that carried out counseling on physical activity based on the 5A model, given the plurality of theories and models addressed in previous reviews, and for guiding more specific advances for future studies.

## 5. Conclusions

Based on data from four interventions conducted in samples of adults, except for increasing the daily number of steps, counseling based on the 5A model did not reflect significant findings in relation to physical activity indicators. Given the potential of the 5A model, future studies are recommended with a better description of the strategies, as well as a more robust methodology, to strengthen the evidence.

## Figures and Tables

**Table 1:** Descriptive characteristics of the studies included in the synthesis (*n* = 4).

Reference	Country	Mean Age (% of Women)	Sample Characteristics	Main Objective
Galaviz et al., 2017 [22]	México	49 (77)	Adults who do not meet physical activity recommendations, but without clinical impediments.	Increase the use of the 5A model in counseling and observe whether the intervention provided an increase in users’ physical activity.
Reed et al., 2019 [23]	United States	48 (80)	Inactive adults assisted by a primary health care unit located in a rural setting.	Test the use of the 5A model in counseling to increase physical activity.
Viglione et al., 2019 [24]	United States	55 (33)	Veterans with a BMI ≥ 30 kg/m^2^ or between 25 and 29.9 km/m^2^ diagnosed with a comorbidity (hypertension, obstructive sleep apnea, high cholesterol, prediabetes, and metabolic syndrome).	Determine the feasibility and acceptability of a technology-based method compared to usual care. Test the impact of this method on weight, diet, and physical activity.
Wang et al., 2015 [25]	China	40 (65)	Adults with chronic insomnia.	Verify the effects of a counseling-based intervention on physical activity and sleep restriction.

**Table 2:** Methodological characteristics of the studies included in the synthesis (*n* = 4).

Reference	Study Design	Professionals Who Implemented the Intervention	Study Protocol
Galaviz et al., 2017 [22]	Effectiveness-implementation hybrid study.	General practitioners working in the public health service.	Previously trained physicians offered counseling based on the 5A model in regular consultations.
Reed et al., 2019 [23]	Randomized Controlled Trial.	Nurses and Researchers.	Elaborating action plans to improve self-regulation and sending motivational weekly text messages.
Viglione et al., 2019 [24]	Randomized Controlled Trial.	Health professionals and students.	Delivery of educational material and counseling (face-to-face and remote).
Wang et al., 2015 [25]	Randomized Controlled Trial,	Psychologists.	Offer of four weekly counseling sessions, adjustment of prescription and sleep restriction strategy.

**Table 3:** Physical activity indicators, assessment, and results of the included studies (*n* = 4).

Reference	Tools Used to Assess Physical Activity	Intervention Group	Control Group	Physical Activity Indicator (Time in Which the Assessment Took Place)	Result
Galaviz et al., 2017 [22]	Questionnaire GLTEQ	228 *	231	Physical activity score (6 months post-intervention).	Data were not statistically significant (numbers were not presented in the report).
				Number of people classified as physically active (6 months post-intervention).	Data were not statistically significant (numbers were not presented in the report).
Reed et al., 2019 [23]	Questionnaire GLTEQ; Fitbit Charge 2	29	30	Assessment by questionnaire (4 months).	8.1 (95%CI = 0.1; 16.1)
				Weekly number of steps (4 months).	1266 (95%CI = −520; 3052)
				Active weekly minutes (4 months).	42 (95%CI = −102; 186)
Viglione et al., 2019 [24]	PaffenbargerPhysical Activity Questionnaire	21	22	Self-efficacy for physical activity (3 months).	−1.1 (95%CI = −6.8; 4.7);
				Self-efficacy for physical activity (6 months).	2.1 (95%CI = −4.5; 8.8)
				Self-efficacy for physical activity (12 months).	−2.2 (95%CI = −9.1; 4.1)
Wang et al., 2015 [25]	IPAQ Long version	35	36	Daily number of steps (4 months).	2231 (95%CI = 474; 3987)

*: Unprompted group; GLTEQ: Godin’s Leisure-Time Exercise Questionnaire; 95%CI: 95% confidence interval.

## Data Availability

All unpublished data of interest can be requested from the corresponding author.

## Supplementary Materials

The following supporting information can be downloaded at: https://www.mdpi.com/article/10.3390/bs13060476/s1, File S1: The full presentation of the systematic searches.
