# Supplementary material for: Effectiveness of the 5A Counseling Model-Based Interventions on Physical Activity Indicators in Adults: A Systematic Review
Source: Behav Sci (Basel). 2023 Jun 6;13(6):476. doi: 10.3390/bs13060476 (PMC10295221; doi:10.3390/bs13060476)
Supplement: Supplementary file 1 [file behavsci-13-00476-s001.zip › behavsci-2412853-supplementary.pdf]

Supplementary Material File S1. Systematic searches used in electronic databases (date: 16 May 2022).

**Database**	**Syntax**	**Number**
Embase	('physical activity' OR exercise OR walk*) AND (5a OR 5as)	572
Lilacs	(I) English: "5A" AND "physical activity"<br>(II) Portuguese: "5A" AND "atividade física"	6
Pubmed	(((physical activity[Text Word]) OR (exercise[Text Word])) OR (walk*[Text Word])) AND (((5A[Text Word]) OR (5As[Text Word])) OR (5A's[Text Word]))	112
Scielo	(I) English: (((5A) OR (5As) OR (5A's)) AND (("physical activity") OR (exercise) OR (walk*)))<br>Filters: (Collections: Brasil)<br>(II) Portuguese: (((5A) OR (5As) OR (5A's)) AND (("atividade física") OR (exercício) OR (caminhada)))<br>Filters: (Collections: Brasil)	4
Scopus	(TITLE-ABS-KEY ((("physical activity") OR (exercise) OR (walk*))) AND TITLE-ABS-KEY (((5a) OR (5as) OR (5a's))))	199
Sportdiscus	(("physical activity") OR (exercise) OR (walk*)) AND ((5A) OR (5As) OR (5A's))	31
Web of Science	((TS=("physical activity")) OR TS=(exercise)) OR TS=(walk*) AND ((TS=(5A)) OR TS=(5As)) OR TS=(5A's)	213
